# Early detection and personalized treatment in oral cancer: the impact of omics approaches

**DOI:** 10.1186/s13039-016-0293-1

**Published:** 2016-11-23

**Authors:** Ilda Patrícia Ribeiro, Leonor Barroso, Francisco Marques, Joana Barbosa Melo, Isabel Marques Carreira

**Affiliations:** 1 Cytogenetics and Genomics Laboratory, Faculty of Medicine, University of Coimbra, Polo Ciências da Saúde, Coimbra, 3000-354 Portugal; 2 CIMAGO - Center of Investigation on Environment Genetics and Oncobiology - Faculty of Medicine, University of Coimbra, Coimbra, 3000-354 Portugal; 3 Maxillofacial Surgery Unit, Coimbra Hospital and University Centre, CHUC, EPE, Coimbra, 3000-075 Portugal; 4 Department of Dentistry, Faculty of Medicine, University of Coimbra, Coimbra, 3000-075 Portugal; 5 Stomatology Unit, Coimbra Hospital and University Centre, CHUC, EPE, Coimbra, 3000-075 Portugal

**Keywords:** Oral cancer, Early diagnosis, Omics data, Molecular biomarkers, Molecular profiling

## Abstract

**Background:**

Oral cancer is one of the most common malignant lesions of the head and neck. This cancer is an aggressive and lethal disease with no significant improvements in the overall survival in the last decades. Moreover, the incidence of oral HPV-positive tumors is rising, especially in young people. This oral neoplasm develops through numerous molecular imbalances that affect key genes and signaling pathways; however, the molecular mechanisms involved in the pathogenesis and progression of oral tumors are still to be fully determined. In order to improve the quality of life and long-term survival rate of these patients, it is vital to establish accurate biomarkers that help in the early diagnosis, prognosis and development of target treatments. Such biomarkers may possibly allow for selection of patients that will benefit from each therapy modality, helping in the optimization of intensity and sequence of the treatments in order to decrease side effects and improve survival.

**Conclusion:**

In this review we discuss the current knowledge of oral cancer and the potential role of omics approaches to identify molecular biomarkers in the improvement of early diagnosis, treatment and prognosis. The pursuit to improve the quality of life and decrease mortality rates of the oral patients needs to be centralized on the identification of critical genes in oral carcinogenesis. Understanding the molecular biology of oral cancer is vital for search new therapies, being the molecular-targeted therapies the most promising treatment for these patients.

## Background

Oral cancer is part of a group of cancers named head and neck cancer (HNC), which enclose a wide set of diverse tumor types arising from various anatomic structures, such as oral cavity, oropharynx, larynx, hypopharynx and nasopharynx. Histopathologically, squamous cell carcinomas (SCCs) represent nearly 90% of all these tumors and over 50% arise in the oral cavity [1]. The most significant and well-established risk factors related to this neoplasm are tobacco use (smoked or chewed), excessive alcohol consumption and/or human papilomavirus (HPV) infection. HPV genomic DNA is present in almost 25% of oral squamous cell carcinomas (OSCCs) [2, 3] and patients with HPV-positive oropharyngeal tumors have a better prognosis compared with HPV-negative oropharyngeal patients, although the reasons for this are not yet complete clarified [4]. It could be related to the more frequent HPV positivity in individuals having no other risk factors and to their usually different histology (poorly differentiated, basaloid) [2].

Worldwide, oral and pharyngeal tumors, when combined, represent the sixth most common cancer, with more than 300,000 estimated new cases and approximately 145,000 deaths from oral cavity cancer (including lip cancer) in 2012 [5]. Furthermore, it is estimated that its incidence will raise due to population growth, aging and the adoption of a lifestyle associated to cancer-risk factors [6]. It is interesting to note that two types of behavior and wariness have apparently contributed in opposite ways: in one way a slight decrease has been observed in the overall incidence of these tumors in the past two decades [2], which is most probably associated with a reduction in the prevalence of smoking [7]; on the other hand, an increase has been detected especially in oropharyngeal cancers (base of tongue and tonsillar) [8], which can be associated with the increase in oral and oropharyngeal HPV infections [7, 9]. Since oral cancer has been more common in men than in women HPV vaccination for both genders should be an option to be considered regarding HNC prevention, but further studies are needed to determine the efficacy and cost-benefit of this kind of vaccination program. Although the median age for head and neck cancer diagnosis is around 60 years, it should be noted that an increase of these tumors has been described in younger patients, i.e. less than 40 years old, mainly with HPV-associated oropharyngeal cancer [10, 11].

Despite technological advances and improvements in OSCC diagnosis and treatment modalities, its 5-years survival rate stays low, around 50–60% [12]. This discrepancy can be explained both by the delay in the diagnosis and by the relatively high tumor recurrence rates detected in these patients. In general, at the time of diagnosis, only one third of OSCC patients have the disease at early-stages (I and II) [13].

The understanding about the biology and pathology of oral carcinogenesis process is still slow. Therefore, a better insight concerning oral cancer proliferation, invasion and cellular migration is clearly needed. This understanding is crucial to search and establish molecular targets to help in the early diagnosis, to predict the prognosis and also to direct the development of new therapeutic strategies. The purpose of this review is to provide a critical analysis of the current knowledge of oral cancer and to discuss the potential translation to clinical practice of molecular signatures using the emergent omic approaches.

### Diagnosis and treatment: clinical features

Diagnosing tumors in the oral cavity might be challenging. The inspection and examination of the oral cavity has an easy access and is simple to perform; however, benign lesions can sometimes mimic the alterations observed in cancer development [14]. Additionally, the potential malignant lesions and cancer in the early stage are very subtle in the clinical appearance, which complicates the early diagnosis and treatment. Moreover, presently, there is no prognostic model devised to accurately predict the risk of OSCC patients recur after the primary treatment. Histopathology is still used as a gold standard for characterization and classification of solid tumors; however, this classification of tumors is often not satisfactory to predict the clinical outcome in individual cases [15]. The disease stage attributed at the diagnosis remains the most important prognostic indicator for OSCC. Thus, the 5-year survival rate for cancers in stage I is almost 80%, while for stage IV cancers, this rate decreases to almost 20% [16].

The first-line treatment for OSCC has been surgery, which may be followed by chemo/radiotherapy. Surgery is a debilitating and considerably morbid procedure that impairs the patients’ quality of life as it implies drastic alterations in chewing, swallowing, speech or facial esthetics [17]. A great dilemma for physician is delimiting the exact area of excision [18], because the presence of the tumor in or close to the surgical margin might be indicative of the risk of relapse, which influences the decision of additional treatment. Unfortunately, relapses have been observed in patients with histological tumor-free margins after surgery.

The use of Cetuximab, a monoclonal antibody targeting the extracellular ligand binding domain of the epidermal growth factor receptor (EGFR), combined with radiotherapy is approved for the treatment of locoregionally advanced HNC [19], and for the treatment of recurrent or metastatic disease in combination with platinum-based chemotherapy [20]. The introduction of Cetuximab (Erbitux^®^) in the clinical practice was a great advance in the field of targeted drugs. However, not all patients treated with Cetuximab respond well to therapy due to primary or acquired resistance, limiting significantly the clinical benefit of this drug [21]. Besides Cetuximab there are other molecular targeted agents that have been investigated for OSCC therapy.

The introduction of immune checkpoint inhibitors into HNC treatment has been explored, being the cosignaling of the programmed cell death protein 1 (PD-1) and its ligand (PD-L1) one of the major pathways of interest. PD-1 is a co-inhibitory receptor, since the ligation of PD-L1 or PD-L2 to PD-1 inhibits T-cell proliferation and down regulates expression of the anti-apoptotic molecule Bcl-xL, cytokine expression and the mTOR pathway in immune cells [22–24]. Currently, there are several anti-PD-1 and anti-PD-L1 monoclonal antibodies in clinical trials, which presenting early promising results regarding survival benefit in metastatic/recurrent HNC [25]. Due to the manifold tumor evasion strategies and different response rates for treatments, combinational therapies are crucial to treat oral cancer. Thus, adding immunotherapeutics to standard Cetuximab-containing regimens is under investigation [26].

An apparently problem in this field of targeted therapies seems to be the identification of biomarkers that help in the selection of eligible patients to benefit these expensive treatments [27], which reinforce the need of well designed studies in order to select the patients and therefore the drug according to their molecular profile.

### Omics in oral cancer

Emerging OSCC omics data hold great promise for understanding of the oral carcinogenesis process, predicting prognosis and helping in the development of targeted therapies. Advances in the powerful omics technologies: genomics, epigenomics, transcriptomics, proteomics, metabolomics and lipidomics are opening new routes towards biomarker discovery in order to allow early diagnose and to differently discriminate the behavior of each tumor, providing personalized therapies for the patients. Considering the multifactorial nature of OSCC it is mandatory to combine different biomarkers that could lead to the implementation of prophylactic screening programs, decreasing the incidence and mortality and increase the quality of life of these patients. It is important to note that in omics technology, bioinformatics tools are required to extract relevant information from the complex huge amount of data and identify players in cancer pathways. Indeed, in this post-genomic era several questions remain to be addressed. The central question for oral oncology research seems to be the following: which alterations are likely to occur early in oral cancer, and how do these molecular imbalances thus affect the clinical outcome of the patients? It is very difficult to explain the different outcomes for patients with the same clinical disease submitted to the same treatment. In this way, integrative molecular research in larger cohorts is imperative to better understand this devastating disease. Additional questions are also important to be considered, namely: are clinicians up to date on pharmacogenomics to apply these data in their clinical choices? Can society afford personalized medicine at all, considering the actual costs related to high throughput technologies and targeted treatments?

### Genomic, epigenomic and transcriptomic profiling

Oral cancer development lies in stepwise accumulation of genomic and epigenomic changes, compromising cell division, differentiation, immune recognition, tissue invasion and metastasis [28]. DNA alterations can lead to aberrant RNA and protein, with widespread deregulation of transcription during oncogenesis. High-throughput technologies have been used to try shed light on these driving molecular mechanisms and ultimately improve the lives of OSCC patients.

Genomic changes play a significant role in tumourigenesis as well as in inter- and intratumour heterogeneity [29]. In OSCC, alterations in almost all chromosomes have been frequently reported. Nevertheless, some chromosomal regions are described in the literature as consistently altered in these tumors. Tumor progression models have been constructed, being Califano and colleagues in 1996, the first to describe a progression model for OSCC, where losses at chromosomal regions 3p, 9p, and 17p are considered early events in the carcinogenesis process [30]. Our group showed frequent copy number gains at 3q, 6p, 8q, 11q, 16p, 16q, 17p, 17q and 19q, and copy number losses at 2q, 3p, 4q, 5q, 8p, 9p, 11q and 18q [31–33], being mapped in these regions several genes related with proliferation and cell cycle regulation. Gain in the 3q26 region, which contains the *hTERC* and *SOX2* genes, is common in all squamous cell carcinomas and their detection could be important for early detection and for accurate prognosis of these patients [34]. We verified that gains of *MYC* and *WISP1* genes seem to suggest higher propensity of tumors localized in the floor of the mouth [35]. However, much regarding the genomic characterization of oral cancer is still unknown, as only a few genes associated with this disease have been identified, and without implementation in routine clinical practice. The application of Next-Generation Sequencing (NGS) technologies has resulted in systematic efforts to characterize the mutational spectrum, genomic alterations, and clonal evolution of these tumors. Initial whole exome sequencing studies performed by Stransky and colleagues [36] and Agrawal and colleagues [37] identified commonly-mutated genes in HNC. This was expanded upon with the Cancer Genome Atlas (TCGA), which generated an integrated genomic annotation of molecular alterations using a comprehensive multi-platform characterization of head and neck squamous cell carcinomas to detect somatic variants [38]. Nowadays, core dysregulated pathways and actionable targets are already identified, which may justify the use of more focused and cost-effective sequencing panels that potentially yield the same information with less noise [39]. The presence of molecular heterogeneity is a problem for the genomic approaches, since tumor develop through different subpopulations of cells with different levels of genomic imbalances; therefore, the biopsy tissue analyzed might not be representative of all tumor imbalances at all the time. Moving forward, single cell sequencing will opens the door for the study of tumor heterogeneity, revealing cancer clonal evolution [40]. It might allow accurate genome-based diagnosis and consequently specific treatment for each patient with cancer.

Identification of epigenomic biomarkers has so far focused mainly on DNA methylation, since this is a well-known epigenetic phenomenon with an established role in cancer. Growing evidence supports promoter CpG island hypermethylation as a *bona fide* mechanism for gene inactivation. New high-throughput methodologies provide information to characterize methylated sequences at single-base resolution on a genome-wide scale. Recent studies focused on identifying biomarkers of early disease detection, since DNA methylation is considered an early event in cancer process [41]. We found that *WT1* gene promoter methylation is a predictor of a better prognosis and that *MSH6* and *GATA5* gene promoter methylation serve as predictors of a worse prognosis and a shorter survival rate in OSCC [42]. Moreover, we identified *PAX5* gene promoter methylation as associated with tongue tumors [42]. Epigenome-wide analysis confirmed a distinct epigenetic signature in HPV-positive HNC when compared to HPV-negative tumors [43]. Moreover, Pierini et al. described that traditional risk factors (tobacco and alcohol consumption) affect epigenetic modification of specific genes in laryngeal carcinomas, underlining the implication of *CDKN2A* and *MLH1* promoter methylation in invasion of lymph nodes with cancer cells [44]. DNA methylation patterns seem also to be useful to determine whether a second tumor represents a recurrence of the original malignancy or a second primary cancer [45]. Considering the stable, potential reversible nature and variable levels of epigenomic modifications in OSCC, tumor methylation profiling during treatment could provide important insights regarding the treatment efficacy, emphasizing the importance of repeatedly monitoring these patients. An attractive approach to do this monitorization is using biofluids. Several studies evaluated the presence of DNA hypermethylated in serum/plasma, saliva and exfoliated cells with promising results [41, 46].

Different genomic expression in HNC samples compared with normal tissue suggests that gene expression signatures can be used as a predictor of outcome in these patients. Thus, large-scale transcriptome analysis in HNC allowed the categorization of these tumors in four distinct subtypes with differences in recurrence-free survival [47, 48]. Furthermore, gene expression approaches revealed that over-expressed DNA repair genes were linked with reduced therapeutic response to radiotherapy, which can be a suitable predictor of post-radiotherapy clinical outcome [49]. Transcriptomic expression profiling confirmed the presence of molecular heterogeneity within OSCC, highlighting differences between anatomic localizations and among samples belonging to the same TNM stage [50]. Additionally, this kind of data allowed the development of prediction models in order to try identify the risk of local recurrence at surgical margins and of second primary tumors, being needed further independent studies [51].

microRNAs (miRNAs) are a group of small RNAs that specifically modulate gene expression with a significant role in tumorigenesis. Several miRNAs have been found to be up or downregulated in OSCC [52]. A molecular classification of OSCC based on 61 miRNAs with an accuracy of 93% was achieved [53]. Moreover, in this study HPV infection leads to alteration of some miRNAs, which may provide a mechanism for the distinct clinical behavior of HPV-infected tumors. The question of whether circulating miRNAs could be a biomarker for OSCC has been raised. In 2011, Wiklund and colleagues reported some candidate OSCC deregulated miRNAs with associated DNA methylation patterns, and normal miRNA expression in healthy oral epithelium and stroma [54]. These authors showed that OSCC specific miRNA and DNA methylation patterns can be detected and distinguish between patient and controls using oral rinse and saliva. Furthermore, abnormal expression of several long non-coding RNAs (lncRNAs) were associated with tumorigenesis and metastasis in OSCC [55].

### Proteomic, metabolomic and lipidomic profiling

Biochemical and biological techniques have been used to identify proteins, metabolites and lipids in OSCC.

Nowadays, functional proteomics comprise a high-throughput analysis of both basal and phosphorylated proteins active in carcinogenesis process, allowing the assessment of cellular whole protein complements in tissues or the secreted proteins in the biofluids (secretome), such as serum, plasma, urine or saliva [56]. Studies regarding OSCC have shown alteration in proteins associated to cell metabolism and structure, cellular adhesion or cell motility, signal transduction and oncoproteins [57, 58]. The proteomics tools currently available enable the analysis of a huge variety of cellular protein characteristics, namely relative protein quantities, protein cellular sub-localization and their post-translational modifications, allowing the identification of numerous candidate protein biomarkers [59]. However, it is still needed to prove their clinical value. In 2011, He and colleagues discriminate OSCC tumor samples from potentially malignant lesions, such as oral leukoplakia using proteomic patterns [60]. Potential salivary biomarker peptides with sensitivity up to 90% and specificity of 83% in detecting OSCC have also been proposed [61]. Moreover, classification algorithms based on differently expressed serum proteins seems to be able to distinguish patients with HNC from healthy controls with a high degree of sensitivity (83.3%) and specificity (90%) [62].

Metabolite profiling of tissue and biofluid samples from HNC using mass spectrometry-based metabolomic analysis has been demonstrated to be capable of discovering potential biomarkers for disease detection and treatment monitoring [63]. In serum, levels of numerous metabolites linked to the glycolytic pathway, such as glucose, were observed to be higher in oral cancer patients who had disease relapse. Similarly, the levels of some amino acids were shown to be lower in this biofluid [64]. Detection of these altered metabolites in serum of OSCC patients may contribute to early detection of recurrence [65]. In contrast to serum, the levels of metabolites associated with the glycolytic pathway seem to be lower in HNC tissue than in the non-tumor tissues. Likewise, the levels of amino acids are higher in tumor tissues [64]. Analogous results have also been previous demonstrated in colon and stomach cancer [66]. In 2015, Hu and colleagues demonstrated that the metabolic phenotypes are distinct between high and low invasive HNC cells as well as between stem-like oral cancer cells and non-stem cancer cells [67].

The alterations in lipid profile have also long been associated with cancer, since lipids play a key role in maintenance of cell integrity; however, few reports are available on plasma lipid profile in HNC. An inverse relationship was found between the lipid levels and occurrence of oral cancer, thus, lower plasma lipid status may be a useful indicator of neoplastic lesions [68, 69].

### Omics data integration

Technological advances in high throughput microarrays, DNA sequencing, RNASeq methods and mass spectrometry approaches, together with computational and algorithmic progress, have generated progressively large amount of omics data accessible in public databases. The main problem in comparing results of data-sets generated by different research groups is the use of different platforms and also differences in sample preparation, being the correlations sometimes disappointing. The integration of omics data can yield more than the sum of the individual omics experiments, displaying the interactions that can occur among all classes of molecules in a cell, determining the cellular physiology and behavior [70], which seems to be vital to fulfill the dream of individualized cancer care and treatment.

### Molecular data to early diagnosis and to establish biomarkers of prognosis in OSCC

When patients are treated within one month of onset of the symptoms, they have a 5-year survival rate of 86%, but this decreases to 47% at seven months after the beginning of the symptoms, and there is very scarce possibility of survival if the treatment occurs only after twelve months [71]. The nonspecific clinical appearance of dysplastic and early malignant lesions further highlights the need to develop objective methodologies for earlier detection [72]. Thus, taking into account that the molecular alterations may occur before phenotypic ones, molecular profiling holds great promise to predict disease progression. Simultaneously, to achieve a significant improvement in the diagnosis it is important to use the great advantage of this neoplasm, which is the accessible localization of the tumors, allowing frequent examinations, patient monitoring and easy acquisition of samples. In order to follow up the patients, biopsies do not gather all the needed requisites, since it is questionable and unpractical to get biopsies from multiples sites and/or during short periods of time, as well as from all different kinds of morphologically suspicious tissues, due to the discomfort for the patient. Thus, it seems mandatory to develop a non-invasive or minimally invasive methodology, which combined with the identification of the most appropriate prognostic molecular markers, will be very helpful for the screening of risk populations. This non-invasive methodology will also be helpful for the follow-up of the OSCC patients already undergoing treatment. In this sense, biofluids emerge as a good material to detect/analyze nucleic acid and proteins from the cancer cells, without resorting to biopsies.

## Conclusions

In the present review, we focused on the contribution of genomic, epigenomic, transcriptomic, proteomic, metabolomic and lipidomic studies to the understanding and identifying of biomarkers capable of predict the clinical behavior of oral tumors. These omics approaches will also play an important role in understanding relapses at the primary site after resection, distant metastasis and the development of second primary tumors. However, many questions in this field remain to be answered. The pursuit to improve the quality of life and decrease mortality rates of the oral patients needs to be centralized on the identification of critical genes in oral carcinogenesis. This will enable to choose the treatment strategy to individualized therapy based on the molecular patient’s profile. Besides the diagnosis of oral cancer, the molecular analysis will also make possible the monitoring of suspicious lesions in the higher risk populations. In this line, saliva could become in a near future a vital element in the early detection and screening of oral cancer. It will allow performing easy and non-invasive routine health screening of the high risk populations, such as individuals with HPV infection and heavy tobacco and alcohol consumption. It is also important to improve oral health care education and raise the awareness of the general population to this severe disease, which nonetheless has a favorable prognosis when early detected. Understanding the molecular biology of oral cancer is vital for search new therapies. The molecular-targeted therapies represent the most promising treatment for OSCC. EGFR-targeting agents have shown efficacy for treatment of these patients. Several studies including immunotherapeutic strategies and inhibitors of VEGFR, IGF-1R, PI3K/AKT/mTOR and MET have been conducted, being expected in a near future a shift in the management of oral cancer patients towards more personalized treatment.
